# Intestinal Epithelial Barrier Dysfunction in Food Hypersensitivity

**DOI:** 10.1155/2012/596081

**Published:** 2011-09-08

**Authors:** Linda Chia-Hui Yu

**Affiliations:** Graduate Institute of Physiology, National Taiwan University College of Medicine, Suite 1020, no. 1 Jen-Ai Road Section I, Taipei 100, Taiwan

## Abstract

Intestinal epithelial barrier plays a critical role in the maintenance of gut homeostasis by limiting the penetration of luminal bacteria and dietary allergens, yet allowing antigen sampling for the generation of tolerance. Undigested proteins normally do not gain access to the lamina propria due to physical exclusion by tight junctions at the cell-cell contact sites and intracellular degradation by lysosomal enzymes in enterocytes. An intriguing question then arises: how do macromolecular food antigens cross the epithelial barrier? This review discusses the epithelial barrier dysfunction in sensitized intestine with special emphasis on the molecular mechanism of the enhanced transcytotic rates of allergens. The sensitization phase of allergy is characterized by antigen-induced cross-linking of IgE bound to high affinity Fc*ε*RI on mast cell surface, leading to anaphylactic responses. Recent studies have demonstrated that prior to mast cell activation, food allergens are transported in large quantity across the epithelium and are protected from lysosomal degradation by binding to cell surface IgE and low-affinity receptor CD23/Fc*ε*RII. Improved immunotherapies are currently under study including anti-IgE and anti-CD23 antibodies for the management of atopic disorders.

## 1. Introduction

Food allergy is reported in 6–10% of the pediatric population and is more frequent in children than adults [1, 2]. The most common allergens include cow's milk, eggs, peanuts, and seafood. Although some types of food allergy remit spontaneously during the first few years of life, it is often associated with later development of extraintestinal allergies that manifest in the respiratory tract and skin [1, 2]. 

The sensitization phase of allergy is characterized by increased IgE synthesis and Th2-type cytokine (IL-4, IL-5, and IL-13) responses. Elevated production of IL-4 by mononuclear cells has been demonstrated in the blood and intestinal mucosa of atopic individuals [3, 4]. IL-4 induces germ line *ε* transcription for isotype class switching in B cells and promotes B cell proliferation to increase the synthesis of antigen-specific IgE [5]. In addition to its presence in serum, elevated levels of allergen-specific IgE are detected in the intestinal fluid and stool samples in food-allergic patients [6–8]. The presence of IgE in the gut lumen was also observed in parasitic infection animal models infected with parasites [9]. The binding of IgE to the high affinity Fc*ε*RI on the surface of mast cells is the hallmark of allergy. Cross-linking of IgE by specific antigen induces mast cell degranulation and release of mediators, thereby, causing anaphylactic responses [10]. Anaphylactic reactions in food allergy are associated with enhanced epithelial ion transport with passive outflux of water which is responsible for clinical diarrheal symptoms [11, 12]. The release of mast cell mediators, for example, histamine, prostaglandin, and serotonin, is involved in the stimulation of epithelial ion secretion [13, 14].

## 2. Immunopathogenesis of Intestinal Sensitization: Role of Bacterial Products and Intestinal Epithelial Cells

A number of factors are involved in the onset of food sensitization, including genetic traits, allergen exposure, and environmental stimuli. Other factors that affect the outcome of allergic diseases include the age at which food antigen is introduced, formula versus breast-feeding, dietary composition, and gastrointestinal infection status. Recent evidence has implicated a critical role of intestinal microflora in the developmental stage of food allergy. In healthy individuals, the colonic lumen hosts over 100 trillion commensal bacteria, the microfloral composition of which is established during the neonatal period by exposure to vaginal microbes through the birth canal or to bacteria of the digestive tract of the mother via food ingestion [15, 16]. Commonly identified enteric commensal bacteria include those in the phyla Firmicutes (species such as *Lactobacillus*, *Clostridium*, *Enterococcus*), Bacteroidetes (species such as *Bacteroides*), Proteobacteria (species such as *Escherichia coli*), and Actinobacteria (species such as *Bifidobacteria*) [17, 18]. These bacteria are traditionally viewed as cohabitant organisms in the gut that only require elimination in cases where abnormal translocation to systemic blood or extraintestinal organs occurs. It was only recently that our coevolved microorganisms have started to be viewed in a more positive light, with more and more evidence of their beneficial effects to the host [16, 19]. It is now generally believed that the enteric microbiota is involved in the regulation of multiple physiological functions in the gastrointestinal tract. These include competition with and the reduction of pathogen colonization, the degradation of nondigestible dietary substances, the production of short chain fatty acids, folic acids and vitamins, and the stimulation of normal epithelial turnover, as well as the shaping of the mucosal immunity [16, 19–21]. 

Evidence gathered in germ-free and gnotobiotic mouse models led to the identification of another beneficial role of gut bacterial flora, the induction of oral tolerance. The term “oral tolerance” has been defined as a systemic immune unresponsiveness to a specific antigen that had been previously administered via the oral route. The breakdown of oral tolerance has been suggested to be involved in the pathogenesis of food allergy. In contrast to conventionally raised animals, germ-free mice do not generate immune tolerance against fed antigens [22, 23]. The Th1-mediated responses such as the production of IgG2a and IFN*γ* were abolished while the Th2-mediated synthesis of IgE, IgG1, and IL-4 remained high in germ-free mice orally administered ovalbumin as a tolerogen before a systemic challenge with the same protein [22]. Interestingly, oral tolerance may be restored in germ-free mice by inoculation with a single strain of commensal bacteria such as *Escherichia coli* or *Bifidobacterium infantis* [23]. Moreover, mice given oral antibiotics that cause commensal depletion during infancy displayed increased plasma levels of IgG1 and IgE, and decreased IgG2a, in parallel with enhanced IL-4 secretion in stimulated spleen cells [24]. The polarized Th2 immune responses in antibiotic-treated mice were reversed by supplementation with *Enterococcus faecalis*, and to a lesser extent with *Lactobacillus acidophilus* [25]. These findings underscore the role of intestinal commensal bacteria in the induction of oral tolerance and the prevention of allergy. 

Signaling receptors activated by microbe-associated molecular pattern (MAMP) may regulate the host susceptibility to food allergy. Polymorphism of CD14, the binding receptor for lipopolysaccharide, has been associated with the development of nonatopic asthma and food allergy [26–28]. However, other studies found no evidence of gene polymorphism of CD14, toll-like receptor (TLR)-2 and -4 in food allergic diseases [29, 30]. Another study demonstrated increased production of tumor necrosis factor-alpha and interleukin-1 in cord blood mononuclear cells upon TLR2, TLR4, and TLR5 activation in newborns who later develop allergic diseases, suggesting a link between heightened perinatal TLR response and allergy development [31]. Using animal models lacking functional TLR4 in C3H mice background, it was demonstrated that TLR4-dependent signals provided by intestinal commensal bacteria inhibit the development of allergic sensitization, including Th2-skewing responses and anaphylaxis to peanut allergens [32, 33]. It is worth noting that both intestinal epithelial cells and lamina propria macrophages express CD14 and TLR4 at variable levels that change in intestinal inflammation [34, 35]. The role of MAMP signaling by epithelial cells and/or innate immune cells in the mechanism of allergic sensitization is still poorly understood. 

The putative concept underlying development of oral tolerance is that feeding antigen at a high dosage results in clonal deletion or anergy of specific T cell clones in a process that involves Fas/FasL-dependent apoptosis, whereas low antigen dosage favors the pathway of active suppression following the induction of regulatory T (Treg) cells [36]. The different means of tolerance induction are not mutually exclusive but may overlap. Different subsets of dendritic cells have been described in the mouse intestine based on their expression of surface molecules such as CD11b, CD11c, CD103, CX_3_CR1, and CD70; these subsets have their own functional specialization that are crucial for determining the induction of immunity or tolerance to gut antigens [37]. For example, certain subtypes of dendritic cells are involved in the differentiation of Th1, Th2, and Th17 cells, or are required for isotype switching of IgA in B cells [37–40]. On the other hand, tolerogenic CD103(+) dendritic cells isolated from the lamina propria or mesenteric lymph nodes drive the development of Treg cells that are crucial for the induction of oral tolerance [41, 42]. 

Recent advances have indicated that intestinal epithelial cells play critical roles in promoting the differentiation of dendritic subsets with tolerogenic phenotypes, suggesting that the local microenvironment is important for driving oral tolerance. Recent studies have indicated that epithelial-derived transforming growth factor (TGF)-*β* and retinoic acid were required for the upregulation of CD103 on dendritic cells, and the epithelial-conditioned dendritic cells are in turn capable of inducing the differentiation of adaptive Foxp3+ Treg cells with gut-homing properties [43, 44]. Others have reported that the expression of integrin *α*v*β*6 in epithelial-derived exosomes, when coupled with food antigen, results in the development of TGF*β*-producing tolerogenic dendritic cells that promote active production of TGF*β* in Treg cells [45]. Moreover, a transient break of epithelial barrier caused by ethanol or a *Vibrio cholerae *zonula occludens toxin hexapeptide induced the development of Treg cells through mechanisms that requires the presence of an intact microflora and dendritic cells [46]. These findings suggest that intestinal epithelial cells are involved in the development of tolerogenic dendritic cells and Treg cells that are central for the induction of oral tolerance. 

As feeding of antigen alone leads to oral tolerance, the administration of food proteins with bacterial adjuvants such as pertussis toxin and cholera toxin is used to sensitize animals instead [47–50]. Coadministration of bacterial-derived toxins with antigens was shown to upregulate the expression of MHCII and costimulatory molecules on monocyte- and bone marrow-derived dendritic cells, and to induce a Th2-skewing response with elevated production of IL-4 and increased synthesis of antigen-specific IgE and IgG2a [51, 52]. Accumulating data indicate that bacterial adjuvants may affect the intestinal dendritic cells population. A recent report documented that cholera toxin induce the selective expansion of CD11c(+) dendritic cell subsets and increased maturation of all subsets of dendritic cells associated with upregulated OX40 ligand expression in the mesenteric lymph nodes and consequent promotion of Th2-driven responses. The authors suggest that the OX40L molecule may play a critical role in sensitization to food allergens [53]. Another study indicated that exposure to cholera toxins induces allergic sensitization to peanut extracts by causing a shift of the subsets of dendritic cells in the lamina propria and Peyer's patches with more inflammatory CD11b(+) and fewer tolerogenic CD103(+) cells [54]. Moreover, increased expression of T-cell immunoglobulin and mucin domain molecule (TIM)-4 was found in mouse bone marrow-derived dendritic cells *in vitro* after concurrent exposure to cholera toxins and peanut allergens compared to those treated with allergens alone [55]. Adoptive transfer of these TIM4-expressing dendritic cells sensitizes naïve mice to orally challenged peanut allergens, as evidenced by heightened Th2 cytokine responses and elevated levels of specific antibodies of IgE in the serum and intestinal tissues [55]. The interaction between TIM1 expressed on CD4(+) T cells and TIM4 expressed on dendritic cells has been suggested to play an important role in the polarization of Th2 responses and the inhibition of tolerance development [56, 57]. Similar effects of TIM4 overexpression in intestinal mucosal dendritic cells and intestinal sensitization to ovalbumin were reported after exposure to staphylococcal enterotoxin B as the adjuvant [58]. These findings all point to the regulatory role of intestinal dendritic cells in the determination of the nature of antigen-specific T cell differentiation, and the induction of Th2 skewing caused by bacterial toxins for allergic sensitization to food proteins.

## 3. Intestinal Epithelial Barrier Functions

The luminal surface of the gastrointestinal tract from the stomach to the rectum is covered by a single layer of epithelial cells. The vast epithelial surface of the gut allows for efficient nutrient uptake of energy sources in the individual. However, the epithelial layer must also form a competent line of defense since it is constantly bombarded with noxious luminal contents, such as antigenic substances and pathogens. The epithelial layers are maintained in a dynamic equilibrium governed by the balance between crypt stem cell proliferation and villus/surface cell shedding. The newly proliferated stem cells in the crypt regions differentiate into absorptive and secretive types of epithelial cells with high expression of brush border enzymes and transporters, and concurrently migrate upward to the apex of the villi where cells then undergo apoptosis and detachment [59]. During the differentiation process, epithelial tight junctions (TJs) are formed at the cell-cell contact sites to seal off gaps between cells. This physical barrier constituted by the closely linked epithelial cells is the rate-limiting factor that determines gut permeability. 

**Figure 1:**
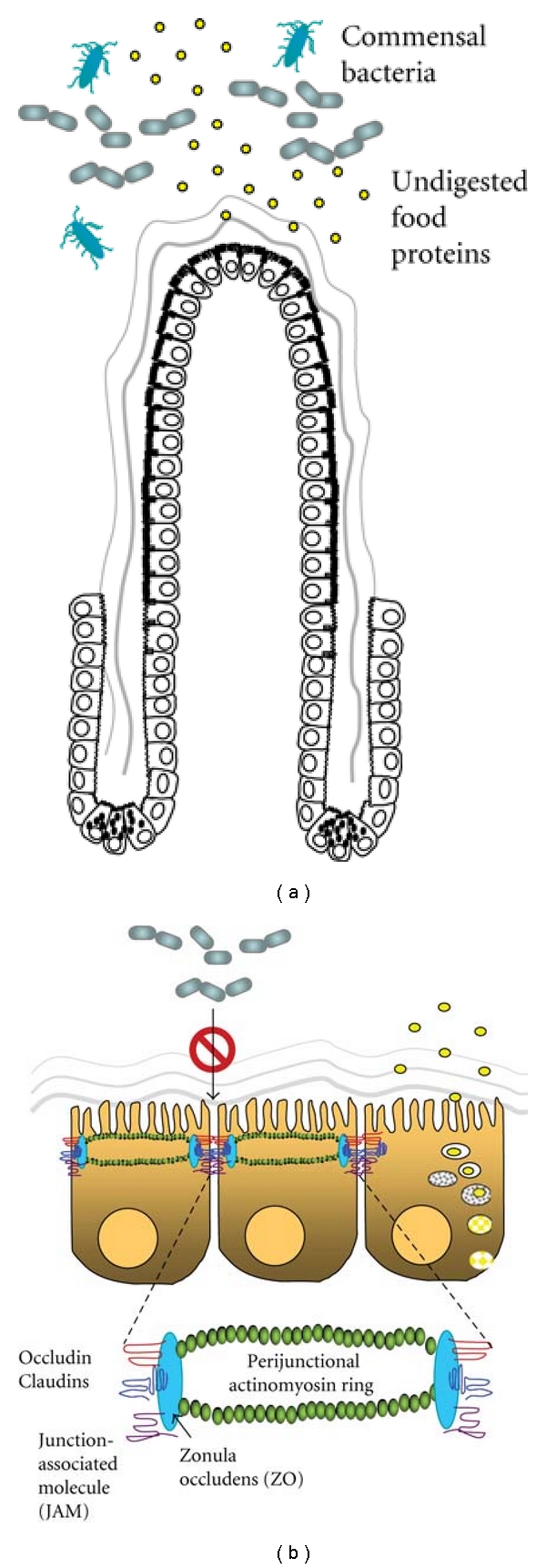
Intestinal barrier functions. (a) Differentiated intestinal villous epithelial cells and the covering mucus layer form a physical barrier to separate luminal contents from the lamina propria. The epithelial barrier prevents the entry of noxious substances, such as undigested food proteins and commensal bacteria, into the body proper. (b) Tight junctional complexes located at the most apical portion of the lateral plasma membrane between two cells excludes the influx of antigenic proteins and bacteria through paracellular routes. The transmembraneous junctional proteins, for example, claudins, occludin, or junction-associated molecule (JAM), are linked to intracellular zonula occludens (ZO) which are bridges to perijunctional actinomyosin rings. Most dietary proteins are digested to small peptides and amino acids before being absorbed into enterocytes via specific transporters. A very small percentage of intact proteins may be endocytosed into epithelial cells but are degraded by lysozymes and lose their antigenic properties. The lysosomal degradation pathway thus prevents the entry of intact proteins through transcellular routes.

The tight junctional complexes forming the most apical portion of the lateral plasma membrane between two cells only allow molecules smaller than about 500 Daltons to cross and exclude the influx of antigenic proteins and bacteria through paracellular routes. The transmembraneous junctional proteins, for example, claudins, occludin, or junction-associated molecule (JAM) are linked to intracellular zonula occludens (ZOs) which are bridges to cytoskeletal actin and myosin filaments [60, 61] (Figure 1). The organization of TJ proteins and perijunctional actinomyosins is regulated by a complex network of signaling pathways. Contraction of the actinomyosin filaments, which opens up paracellular junctions, is mediated by the phosphorylation of myosin light chain via the activation of myosin light chain kinase or Rho-associated kinase [62–64]. In addition to the physical opening of TJs, Rho-associated kinase also mediates the endocytosis of TJ proteins into vacuolar apical compartments [64]. Different isoforms of protein kinase C (PKC) are involved in TJ opening and assembly [65]. The atypical PKCzeta is the sole isoform found at intercellular contact sites [66, 67]. Previous studies have shown that membrane translocation and phosphorylation of PKCzeta leads to decreased transepithelial epithelial resistance and relocation of ZO-1 and occludin in human intestinal T84 and Caco-2 cell cultures following infection with enteropathogenic *E. coli* [68, 69]. Other reports demonstrated that the activation of PKCzeta causes the redistribution of occludin away from the intercellular junctions by direct phosphorylation of this tight junctional protein [70]. Recent *in vivo *data have further supported a critical role of PKCzeta activation in the disruption of TJs and gut permeability increase in bowel obstruction models [71].

Structural damage to TJ proteins may also depend on excessive epithelial cell death in examples of bacterial and parasitic infection, and in metabolic and inflammatory stress. Numerous pathogens including *Helicobacter pylori* [72, 73], enterohemorrhagic *E. coli* Shiga-like toxin [74], *E. coli* lipopolysaccharide [75–77], *Salmonella enterica* [78], *Citrobacter rodentium* [79], and* Giardia spp.* [80, 81] were reported to cause epithelial cell apoptosis. It has been demonstrated that caspases (cellular proteins involved in the apoptotic cascade) may directly cleave TJ proteins [82]. Metabolic stresses, such as mesenteric ischemia/reperfusion and hemorrhagic shock, evoke epithelial cell apoptosis and necrosis that are associated with mucosal barrier dysfunction and abnormal bacterial translocation [83–88]. 

Transcellular transport of particles and proteins is limited by endosomal degradation within enterocytes. Although a small amount of intact protein may be endocytosed into epithelial cells in physiological conditions, most of it is sorted into lysosomal compartments for degradation, and, therefore, transcytosis of whole proteins with antigen properties is normally prevented [89]. An early study showed that less than 3% of proteins remain in their intact bioactive form after luminal-to-basolateral passage across the intestinal epithelial layer [90].

## 4. Epithelial Barrier Defects in Intestinal Allergy

Dietary proteins are mostly digested by gastric and pancreatic proteases, as well as by integral brush border enzymes, and converted to small peptides and amino acids, which are then absorbed by enterocytes via electrogenic or sodium-dependent transporters. Undigested proteins usually do not gain access to the gut lamina propria due to exclusion by the physical tight junctional barrier and intracellular degradation by the lysosomal enzymes. Nevertheless, an apparent defect in epithelial barrier was noted in food allergy. Early clinical studies in children with cow's milk allergy demonstrated intestinal permeability rise after, but not before, allergen challenge [91–93]. A recent study using small intestinal biopsy specimens exposed to food allergen *in vitro* has shown decreased expression of tight junctional protein, that is, occluding, claudin-1 and ZO-1, in tissues obtained from patients with food allergy compared to those from normal subjects after antigen challenge [94]. These studies suggest that allergen challenge in sensitized individuals leads to enhanced intestinal permeability. 

Experimental models indicate that the breach of epithelial barrier may be a consequence of Th2 switching or may possibly reflect exaggerated responses and viscous cycles caused by mast cell activation [10]. Direct effects of type 2 cytokines, for example, IL-4 and IL-13, on the modulation of intestinal epithelial cell permeability have been demonstrated in human epithelial cell cultures [95–97]. Both IL-4 and atopic serum decreased the transepithelial resistance, and selectively increased the apical-to-basal movement of a macromolecular protein, that is, horseradish peroxidase, through both transcellular and paracellular pathways across the human colonic epithelial T84 monolayer [95, 96]. Others reported that IL-4 increased the expression of pore-forming tight junctional protein claudin-2, which correlated with the enhanced epithelial permeability [98]. Recent studies have demonstrated that IL-13 decreased the transepithelial resistance of human colonic epithelial HT29/B6 cells through the induction of cell apoptosis and increased expression of claudin-2 [99, 100]. Moreover, the involvement of phosphatidylinositol 3-kinase has been identified in the signaling pathways of IL-4 and IL-13-increased intestinal epithelial permeability using cell culture models [96, 97]. There is also evidence that mediators released from mast cells, for example, tryptase and tumor necrosis factor (TNF)-alpha, contribute to the increased epithelial paracellular permeability [101–104].

Although the link between enhanced permeability in gut epithelial barrier and food allergy is widely accepted, it is not clear which one happens first during the sensitization phase. A previous study in rats has shown that chronic psychological stress, which increases uptake of luminal proteins, may predispose animals to sensitization of orally delivered antigens [105]. The underlying factors that contribute to gut barrier defects caused by psychological stress include corticotrophin-releasing factor and nerve growth factor, as well as mast cell activation [105–108]. However, psychological stress also modulates mast-nerve cell interaction and increases mast cell-dependent bacterial adherence and uptake in enterocytes as well as follicle-associated epithelium on Peyer's patches [109–111]. Therefore, we cannot rule out the possibility that nerve and bacteria are involved in the stress-induced intestinal sensitization by altering immune predisposition. Another report demonstrated that mast cell-dependent epithelial permeability rise predisposes mice with IL-9 overexpression to oral antigen sensitization. The intestinal sensitization may be prevented by mast cell stabilizer cromolyn that blocks mast cell activity and intestinal permeability [112]. However, there is no direct evidence that intestinal barrier dysfunction is the main initiating factor for intestinal allergic sensitization. The use of antigens with protease activity for disruption of epithelial barrier in experimental models may tease out the order between permeability change and allergic sensitization. A murine model of allergic respiratory inflammation has been recently developed by repeated intratracheal administration of proteolytically active Pen c13, a major allergen secreted by fungal *Penicillium citrinum*, without the use of adjuvant [113]. The protease activity of the allergen and the resultant tight junctional disruption of respiratory epithelial cells were found associated with the development of airway allergic sensitization [113]. To date, a direct role of intestinal permeability rises, and luminal antigen leakage in the sensitization stage of food allergy remains to be established.

## 5. Mechanism of Enhanced Transepithelial Antigen Transport in Allergic Intestines

It is generally accepted that intestinal anaphylactic reactions are caused by biological mediators released from mast cells in the lamina propria after antigen cross-linking of IgE on the cell surface, suggesting abnormal transepithelial transport of luminal antigens in food allergy. An intriguing question then arises: how do macromolecular food antigens cross the intestinal epithelial barrier? Abnormal antigen passage through specialized lymphoid organs, that is, Peyer's patches, in the intestinal tract has been suggested as one mechanism responsible for the lack of tolerance [114–116]. However, in comparison to the limited exposing area of the Peyer's patches, villous epithelium with its much larger relative surface area may play a more important role in the loss of barrier integrity in intestinal allergy.

It was first noticed in rodent models that the addition of antigen to the luminal or serosal sides of the allergic intestine both induces strong ion secretory responses, though with different time frames. Antigen challenge to the serosal side of the intestine induces an immediate increase (~30 sec) in ion secretion, whereas luminal addition of antigen results in a lag phase (~3 min) before the occurrence of the mast cell-mediated epithelial secretory response [13]. The lag phase of the anaphylactic response after luminal challenge appears to reflect the time for antigen transport across the intestinal epithelial cells to activate the underlying mast cells in the lamina propria [13].

Abundant studies exist that show enhanced transcytotic rates of intact proteins across the intestinal epithelium in experimental allergy [117, 118]. Using rat models of food sensitization, the phenomenon of increased antigen uptake within the endosomal compartment was observed in jejunal enterocytes before the occurrence of mast cell activation [48, 117, 119], suggesting that heightened apical-to-basolateral transcellular transport of allergen is mast cell independent. This notion was confirmed in studies using sensitized Ws/Ws rats (mast cell deficiency due to the mutation of the gene c-kit) and mast cell stabilizing agents [119]. The uptake of antigen appeared to be specific and the transport pathway was exclusively transcellular within the first 2 min after challenge. This period of specific transcellular antigen transport before mast cell activation was termed phase I [48, 119]. The period following mast cell activation, as evidenced by an epithelial ion secretory response, was denoted phase II. During phase II, antigens were visualized not only inside endosomes but also within the tight junctions and paracellular regions between enterocytes in allergic animals [47, 119]. The electrical conductance (measurement of ionic permeability through the paracellular pathway) in intestinal tissues of allergic rats was comparable to that of nonsensitized control animals during phase I, suggesting that gut paracellular permeability was not modified in response to sensitization *per se*. Moreover, a gradual time-dependent increase in tissue conductance corresponds to the phenomenon of enhanced paracellular antigen transport in allergic rats during phase II. The abnormal paracellular epithelial permeability in phase II was absent in allergic mast cell-deficient Ws/Ws rats, suggesting a crucial role of mast cell activation in the induction of tight junction opening and increase of paracellular influx that was not antigen specific.

The phenomenon of enhanced transepithelial antigen transport prior to mast cell activation is specific for the allergen to which the rodents are sensitized, suggesting an immunoglobulin recognition mechanism at the epithelial level [117, 118]. Accumulating evidence suggests that a low-affinity IgE receptor (CD23/Fc*ε*RII) may contribute to enhanced antigen recognition and rapid transepithelial transport in allergic animals [11, 47, 48, 90, 120]. CD23 was previously known for its role in regulating IgE synthesis in B cells and promoting B cell proliferation [121–123]. CD23 expression was found in small intestinal epithelial cells in normal and food-allergic humans and rodents, as well as in bronchial epithelial cells in asthmatic patients [48, 118, 124].

**Figure 2:**
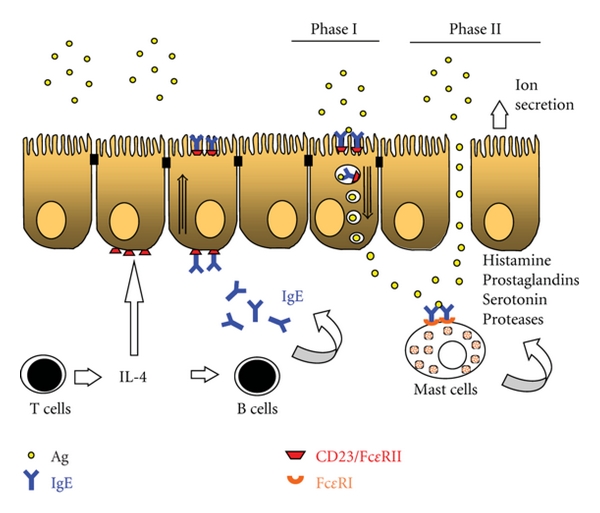
IgE/CD23-mediated transepithelial antigen transport in allergic intestines. In food allergy, Th2-skewing and IL-4 synthesis induce isotype switching in B cells to produce a large amount of IgE that is secreted into serum and gut lumen, or bound to high affinity receptor Fc*ε*RI on mast cell surface. IL-4 also acts on intestinal epithelial cells to upregulate the expression of low-affinity IgE receptor, CD23/Fc*ε*RII. Following exposure to dietary allergens, enhanced luminal-to-serosal transepithelial antigen transport is mediated by IgE/CD23 prior to mast cell activation during phase I. Transcytosed allergens reach the subepithelial lamina propria and cause IgE cross-linking on mast cells, resulting in cell degranulation and anaphylactic responses. The release of mast cell mediators, such as histamine, prostaglandin, serotonin and proteases, are known to induce epithelial ion secretion and to increase paracellular epithelial permeability in phase II.

Studies in sensitized rat models have demonstrated the translocation of CD23 from the cell surface to the membrane of allergen-containing endosomes in intestinal epithelial cells, confirming the internalization of CD23 protein upon luminal antigen challenge [118]. Further studies using genetically mutant mouse models provided evidence for the role of IgE/CD23 in mediating enhanced transepithelial antigen transport in allergy [47, 48]. The phenomenon of augmented antigen uptake in allergic enterocytes was completely absent in sensitized CD23^−/−^ mice and IL-4^−/−^ mice. Moreover, the increased transepithelial antigen uptake in allergic wild-type mice was inhibited luminally with neutralizing anti-CD23 antibodies [47, 48]. Passive sensitization of naive mice by injecting immune serum from allergic mice restored the allergic response, but not if IgE was first depleted from serum, confirming the crucial role of IgE in antigen uptake. In addition, IL-4 increased the expression of CD23 transcript and protein levels in murine intestinal epithelial cell cultures, as well as allergic mouse enterocytes [48]. These findings demonstrate that enhanced transepithelial allergen transport is mediated by IgE/CD23 and regulated by IL-4 in food allergy (Figure 2). Recent evidence further supports the notion that food allergens binding to IgE/CD23 are protected from lysosomal degradation in intestinal epithelium, and therefore, intact antigenic forms of the proteins are preserved during transcytosis [120]. It is now clear that IgE/C23 plays a major role in the mechanism of enhanced transepithelial antigen uptake that is responsible for later mast cell activation and anaphylactic responses in experimental models.

Recent studies have indicated that various isoforms of CD23 mediate bidirectional transport of IgE across the epithelium in allergic murine and human intestine. DNA sequencing revealed the presence of classical and alternative CD23*b* transcripts lacking exon 5 (*b*Δ*5*) or 6 (*b*Δ6) in mouse enterocytes, all of which were translated into functional IgE receptors with distinct endocytic properties [47, 125]. Mouse intestinal epithelial CD23*b*Δ*5* mediated apical to basolateral transport of free IgE, whereas classical CD23*b* displayed higher efficiency in the transcytosis of IgE/allergen complexes [47, 125]. Studies using primary human intestinal epithelial cells and transformed cell lines have also shown that CD23 transports IgE in both the mucosal-to-serosal and serosal-to-mucosal directions [126]. Both CD23 isoforms *a* and *b* transfected into human intestinal epithelial cells transcytosed IgE bidirectional; however, CD23*a* transported IgE/antigen complex faster than CD23*b* in the apical-to-basolateral direction [6]. There remains controversy over which isoform of CD23 is expressed in human enterocytes, and further evidence is needed to confirm the role of IgE/CD23-mediated transepithelial transport in human food allergy [6, 126].

## 6. Novel Diagnostic and Therapeutic Developments Targeting CD23

To date, dietary exclusion is still the most effective measure for the prevention of allergic sensitization and anaphylactic responses in high-risk children and adults. The efficacy of delivering allergen via subcutaneous or sublingual routes for symptom alleviation still needs to be confirmed by large-scale blinded, placebo-controlled trials [127]. A therapeutic approach involving the modulation of dendritic cell functions to disrupt their Th2-skewing ability which has been proposed for food allergy and allergic rhinitis [53–55, 128, 129]. Other immunotherapies, such as the use of anti-IgE antibody, have shown some benefits for peanut allergic patients [130]. Moreover, targeting CD23 with monoclonal antibody has been shown to decrease total serum IgE level in ~75% of allergic asthma patients in a phase I clinical trial and was proposed as candidate therapy for treating allergic diseases for some patient subgroups [131]. Additional information is needed to develop safe and effective treatments for food allergy. A better understanding of the molecular mechanism underlying intestinal sensitization and epithelial barrier defects may hasten the development of prophylactic or therapeutic interventions for the management of atopic disorders.
